# Diagnosing and treating epiploic appendagitis: a review of two cases

**DOI:** 10.1093/jscr/rjad156

**Published:** 2023-03-28

**Authors:** Mehdi B Idrissi, Amal Akammar

**Affiliations:** Department of General Surgery, Haut Atlas Hospital, Azilal 22000, Morocco; Department of Radiology, Haut Atlas Hospital, Azilal 22000, Morocco

## Abstract

Epiploic appendagitis (EA) is a rare condition caused by the infarction of the epiploic appendages, small outpouches of adipose tissue found on the outer surface of the bowel wall. EA results in inflammation and is often mistaken for other gastrointestinal disorders, such as diverticulitis or appendicitis. Diagnosis is primarily made through computed tomography scans, with ultrasound and magnetic resonance imaging used less often. Treatment initially involves analgesia with or without anti-inflammatory medication. However, surgery in the form of laparoscopic appendage removal may be required if symptoms persist or worsen. In total, 2 cases of EA are presented, one mimicking appendicitis and the other sigmoid diverticulitis. The purpose of the presentation is to increase awareness of EA as a cause of abdominal pain and to avoid unnecessary surgery.

## INTRODUCTION

Epiploic appendages are defined as small outpouches of adipose tissue on the outer surface of the bowel wall of the large intestine [1]. These protrusions typically measure between 5 mm and 5 cm and are distributed throughout the colon, with around 50 to 100 present [2]. They receive blood supply from one or two arterioles and single venule [3].

The ischemic infarction of these appendages due to torsion or spontaneous thrombus of their central draining vein, causes them to become inflamed, thus resulting in a condition known as ‘Epiploic appendagitis’ (EA) [4].

Also known as appendicitis epiploica, or epiplopericolitis, it is a rare cause of acute abdominal pain and can be often mistaken for diverticulitis, appendicitis or other gastrointestinal disorders [5].

EA has a higher occurrence rate in male patients [6]. They can arise in any portion of the colon but most commonly found in the rectosigmoid colon. Possible risk factors include obesity and grueling exercise [7].

The diagnosis of acute EA primarily depends on cross-sectional computed tomography (CT) scans, with ultrasound and magnetic resonance imaging (MRI) being less commonly used [8].

The treatment of EA initially involves using anti-inflammatory medications such as non-steroidal anti-inflammatory drugs (NSAIDs) and pain relievers like opioids if needed. Surgery in the form of laparoscopic appendage removal may be necessary if the conservative management doesn’t improve symptoms, if symptoms worsen or if complications such as abscess, obstruction or intussusception occur [3].

While there is ample information about the common causes of acute abdomen such as appendicitis, diverticulitis, bowel obstruction, pancreatitis, perforated peptic ulcer, abscess and pyelonephritis. Knowledge about the rarer causes of an acute abdomen, such as acute EA and acute omental infarction is scarce.

For this reason, it is crucial for healthcare providers to be aware of EA as a possible cause for abdominal pain since a delay in diagnosis can lead to prolonged hospital stay, antibiotic usage and surgical intervention.

Here we present two cases of EA, one mimicking an appendicitis and the other a sigmoid diverticulitis.

The purpose of this presentation is to better the knowledge about this condition, to allow better management and help avoid unnecessary surgery.

## CASE PRESENTATION

### First case

We present the case of a 45-year-old female, with a body mass index (BMI) of 31 with no medical or surgical history. She presented to the emergency department with a 2-day history of abdominal pain, nausea and vomiting. The patient reported that the pain was located in the lower left quadrant and was sharp and constant. Physical examination revealed guarding in the left lower quadrant. Laboratory results were within normal limits. A CT scan of the abdomen and pelvis revealed a thickened and inflamed epiploic appendix in the sigmoid colon. The patient was diagnosed with EA and treated with a NSAID. She was discharged from the hospital the following day with improvement of symptoms and a follow-up was scheduled a week after, with total disappearance of the pain.

### Second case

Chief Complaint: A 75-year-old female with a history of surgically removed ovarian cancer one year prior and BMI of 33, presented to the emergency department with sharp pain in the lower right quadrant of the abdomen.

**Figure 1:**
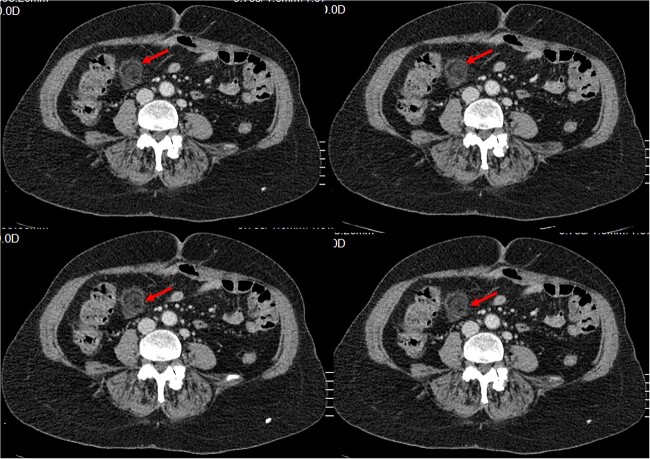
Small intra-peritoneal ovoid fatty mass, well limited by a thin border «Ring Sign/ central dot sign», enhanced after contrast, surrounded by inflammatory changes.

History of Present Illness: The patient reported a sudden onset of sharp pain in the right lower quadrant of the abdomen with no other symptoms. Physical examination revealed tenderness in the same area. Blood work showed an elevated white blood cell count of 13.10 × 1000/𝜇l (4, 8–10) with neutrophilia (87.3%). And C- reactive protein at 45. A CT scan was performed, which showed a typical ‘dot sign’ (Fig. 1), confirming the diagnosis of EA.

Initial Management: The patient was initially treated with antibiotics and opioids, prescribed by another surgeon. After one week of treatment, physical examination showed that the tenderness in the right lower quadrant remained. Blood work was within normal limits.

Treatment Modification: As the patient’s symptoms persisted, antibiotics were immediately interrupted, and the treatment was switched to anti-inflammatory drugs alongside standard analgesia; the patient reported improvement in pain within a few days.

Outcome: The patient’s pain disappeared and tenderness in the right lower quadrant resolved. Follow-up imaging showed resolution of the ‘dot sign’.

## DISCUSSION

EA is a rare condition, that can be very challenging to diagnose. It has an incidence rate of 8.8 cases per million annually [9]. This figure may potentially rise as a result of advancements in diagnostic imaging, and more awareness of this entity.

The specific function of the Epiploic appendage remains to be established. However, it is speculated to potentially function as blood reservoir, aid in immunity and provide protection for the intestinal vessels during colon distention or collapse [3].

Males have a 4-fold greater tendency to develop this condition [10], with other contributing factors including obesity and abnormal physical activity.

EA is typically uncovered as an unexpected result during the examination for other potential sources of acute abdominal pain.

Diagnosing EA can be difficult due to the nonspecific nature of the disease, which can be mistaken for either acute diverticulitis or acute appendicitis. As a result, up to 7% of patients initially diagnosed with acute diverticulitis and 0.3–1% of those diagnosed with acute appendicitis have been reported to have EA [4]. This misdiagnosis can lead to negative consequences such as overuse of antibiotics, hospitalizations and unnecessary surgeries.

Patients with EA often experience a constant, dull pain that is localized and non-migrating in the lower abdomen [3]. This pain is commonly found in the left lower quadrant and may mimic acute diverticulitis [1, 3, 10], as appendages close to the sigmoid colon are often affected. In contrast, appendages near the caecum or ascending colon can cause pain in the right iliac fossa and resemble acute appendicitis [4]. We reported a case of each presentation.

Although most patients with acute EA do not report changes in their bowel habits, a small percentage may experience other symptoms, such as vomiting, bloating, diarrhea and early satiety [10]. Much like our first patient.

Clinicians may mistake EA for other pathological entities such as pelvic inflammatory disease, ureteric stone, gastritis, constipation, ischemic colitis, cancer or enteritis.

EA is usually self-limited, but in rare cases, it may lead to peritonitis, abscess formation, adhesion, bowel obstruction or intussusception [8].

The laboratory results are most commonly within the typical range. However, elevated levels of white blood cells and C- reactive protein (CRP) have been noted [10]. In one of our patients, the only laboratory value of significance was a slightly elevated CRP.

EA lacks pathognomonic clinical and laboratory features. Hence, imaging plays a crucial role in making the proper and accurate diagnosis, and at the same time excluding other potential pathologies.

The diagnosis of acute EA primarily relies on cross-sectional CT, although ultrasound and MRI are occasionally used [6, 8].

In CT imaging, normal epiploic appendages are not easily noticeable. However, they can become visible when they become inflamed or surrounded by ascites. The most common CT feature in acute EA is an oval lesion less than 5 cm in diameter (typical diameter range, 1.5–3.5 cm) that has attenuation equivalent to that of fat, that abuts the anterior colonic wall, and that is surrounded by inflammatory changes, which has recently been referred to in recent studies as the central dot sign [11, 12].

The wall of the colon may be thickened but is most often normal in thickness [8].

According to recent studies, the primary recommended course of treatment for EA is conservative management, either with or without the use of NSAIDs and simple analgesia [3]. Symptoms typically resolve within several days to weeks of conservative management but there is a significant likelihood of recurrence [13].

Antibiotics are not recommended for treatment of EA. In a study by Choi *et al.*, 30 out of 31 patients with EA were treated with antibiotics [14]. The overuse or improper use of antibiotics may occur due to a lack of familiarity among clinicians with EA and the absence of a standard treatment protocol.

Surgery is justified in those who fail with medical management or who exhibit complications such as obstruction, intussusception and abscess formation [8].

In both of our patients, a surgical consult was called upon. Surgery was not recommended as EA remains self-limiting thus a conservative form of treatment was preferred. Both cases evolved positively and rather quickly.

## CONCLUSION

EA, can be difficult to diagnose due to its resemblance to other gastrointestinal disorders. An accurate diagnosis can be made through imaging methods, particularly CT scans and the preferred course of treatment usually involves simple pain management with or without the use of NSAIDs. It is crucial for healthcare providers to have a keen sense of suspicion for EA in patients displaying symptoms of acute abdominal pain and to make a timely diagnosis to avoid any potential complications.

## CONFLICT OF INTEREST STATEMENT

None declared.

## FUNDING

No funding was received for this case report.

## DATA AVAILABILITY

The data that support the findings of this study are available from the corresponding author (MBI) upon reasonable request.
